# Airborne Particulate Matter (PM_10_) Inhibits Apoptosis through PI3K/AKT/FoxO3a Pathway in Lung Epithelial Cells: The Role of a Second Oxidant Stimulus

**DOI:** 10.3390/ijms21020473

**Published:** 2020-01-11

**Authors:** Claudia M. García-Cuellar, Yolanda I. Chirino, Rocío Morales-Bárcenas, Ernesto Soto-Reyes, Raúl Quintana-Belmares, Miguel Santibáñez-Andrade, Yesennia Sánchez-Pérez

**Affiliations:** 1 Instituto Nacional de Cancerología (INCan), Subdirección de Investigación Básica, San Fernando No. 22, Tlalpan, CDMX 14080, Mexico; garcue57@gmail.com (C.M.G.-C.); mobarobiol@yahoo.com.mx (R.M.-B.); qbro@hotmail.com (R.Q.-B.); msantrade@ciencias.unam.mx (M.S.-A.); 2 Unidad de Biomedicina, Facultad de Estudios Superiores Iztacala, Universidad Nacional Autónoma de México, Los Reyes Iztacala, CP 54090 Tlalnepantla, Estado de México, Mexico; irasemachirino@gmail.com; 3 Departamento de Ciencias Naturales, Universidad Autónoma Metropolitana-Cuajimalpa, CDMX CP 05300, Mexico; epigenetics.cancer@gmail.com

**Keywords:** particulate matter, PI3K/AKT/FoxO3A pathway, apoptosis

## Abstract

Outdoor particulate matter (PM_10_) exposure is carcinogenic to humans. The cellular mechanism by which PM_10_ is associated specifically with lung cancer includes oxidative stress and damage to proteins, lipids, and DNA in the absence of apoptosis, suggesting that PM_10_ induces cellular survival. We aimed to evaluate the PI3K/AKT/FoxO3a pathway as a mechanism of cell survival in lung epithelial A549 cells exposed to PM_10_ that were subsequently challenged with hydrogen peroxide (H_2_O_2_). Our results showed that pre-exposure to PM_10_ followed by H_2_O_2_, as a second oxidant stimulus increased the phosphorylation rate of pAKT^Ser473^, pAKT^Thr308^, and pFoxO3a^Ser253^ 2.5-fold, 1.8-fold, and 1.2-fold, respectively. Levels of catalase and p27^kip1^, which are targets of the PIK3/AKT/FoxO3a pathway, decreased 38.1% and 62.7%, respectively. None of these changes had an influence on apoptosis; however, the inhibition of PI3K using the LY294002 compound revealed that the PI3K/AKT/FoxO3a pathway was involved in apoptosis evasion. We conclude that nontoxic PM_10_ exposure predisposes lung epithelial cell cultures to evade apoptosis through the PI3K/AKT/FoxO3a pathway when cells are treated with a second oxidant stimulus.

## 1. Introduction

Air pollution is a problem that mainly affects large cities. It was estimated that in 2016 air pollution was responsible for 4,200,000 premature deaths [1]. Outdoor particulate matter with aerodynamic size ≤ 10 µm (PM_10_) is an important component of air pollution. It is a complex mixture of organic and inorganic compounds, including metals and polycyclic aromatic hydrocarbons, among others. PM_10_ is deposited in the upper respiratory tract, and epidemiological studies have shown PM-induced adverse effects on health such as chronic obstructive pulmonary disease, asthma, fibrosis, and lung cancer. In addition, an increase of 10 mg/m^3^ of PM_2.5_ and PM_10_ was associated with an increase of 8% and 3.4–6% in cancer mortality, respectively [2,3]. Since 2013, PM_10_ has been catalogued as carcinogenic to humans according to the International Agency for Research in Cancer (IARC) [4].

This has led to two measures. The first is at the governmental level, in which there is an urgent demand to follow specific guidelines. For instance, the air quality guidelines from the World Health Organization establish certain limits for PM_10_ exposure (20 μg/m^3^ annual mean and 50 μg/m^3^ 24 h mean). The second measure is the prevailing necessity to understand the cellular mechanism by which PM_10_ exposure is carcinogenic to humans. Therefore, in recent years, a major effort to reveal the possible carcinogenic mechanisms associated with PM exposure has demonstrated that some of those mechanisms include reactive oxygen species (ROS) generation and the consequent oxidative damage to biomolecules, induction of oxidative stress [5], DNA double-strand breaks [6], miRNAs deregulation associated with cell proliferation, autophagy, and DNA damage repair failure [7], and importantly, inadequate chromosomal segregation, among others [8]. In addition, PM_10_ exposure also activates cellular pathways such as extracellular signal-regulated kinases (ERK) leading to cytoplasmic p21(CIP1/WAF1) retention responsible for cytoskeleton remodeling [9], which has been detected during the acquisition of senescence-like phenotype in lung cancer. PM_10_ exposure also activates the STAT3 pathway by Src and PKCζ kinase induction [10] and this pathway is activated for cell survival during oncogenesis and chemoradiotherapy resistance [11,12].

Cell survival and apoptosis evasion are critical mechanisms during carcinogenesis, and, according to the literature, PM_10_ exposure can damage DNA without affecting cell viability [5,6,7,8,10]. Also, PM_10_ exposure might activate other signaling pathways involved in cell survival. In this regard, protein kinase b (PKB or AKT) regulates Forkhead transcription factor box O3a (FoxO3a), which in turn modulates the expression of genes involved in cell-cycle arrest and apoptosis through class I phosphoinositide 3-kinase (PI3K) activation [13]. Therefore, alterations in the PI3K/AKT/FoxO3a pathway could be involved in the inhibition of apoptosis observed in cells exposed to PM_10_. Mediated by cellular receptors, PI3K targets AKT through phosphorylation in Ser 473 (pAKT^Ser473^) and AKT through phosphorylation in Thr 308 (pAKT^Thr308^) by protein mammalian target of rapamycin (mTOR) [14,15]. Then, pAKT^Ser473^ inhibits FoxO3a through phosphorylation in Thr32, Ser253, and Ser315, which promotes the binding of the chaperon proteins 14-3-3, and this triggers FoxO3a translocation to cytoplasm where it can be degraded via proteasome-inhibiting apoptosis [16,17]. In this regard, the inhibition of PI3K signaling by LY294002 compound in primary lung epithelial cells increases the mRNA of caspase 3, which encodes a member of the proapoptotic machinery and downregulates antiapoptotic Bcl-2 mRNA, suggesting that inhibition of this signaling might induce apoptosis [18].

On the other hand, phosphorylation by AKT is not the only posttranslational modification that regulates FOXO proteins; other kinases contribute to regulation of FOXO members, including CDK2, CK1, DYRK1, SGK, and IKK. Additionally, methylation, ubiquitination, and acetylation participate in posttranslational FOXO regulation, while several miRNAs target FOXO at the transcriptional level [19]. Oppositely, hypophosphorylated FoxO3a is required in the nucleus for transcription of proapoptotic proteins such as BIM and PUMA, antioxidant proteins such as catalase and superoxide dismutase 2, and others such as kinase 1 inhibitor protein (p27^kip1^), which is responsible for transition from G0 to G1 in the cell cycle having a role in apoptotic cell death [20].

Based on the above information, we hypothesized that PM_10_ exposure might disturb the AKT/FoxO3a pathway, leading to apoptosis evasion in lung epithelial cells accompanied by a downregulation of some proteins such as catalase and p27^kip1^. Therefore, this study aimed to demonstrate that PM_10_ exposure under low-toxic conditions, followed by hydrogen peroxide (H_2_O_2_) used as a second oxidant stimuli, induces phosphorylation in AKT (pAKT^Ser473^ and pAKT^Thr308^) and pFoxO3a^Ser253^. Using a PI3K inhibitor (LY294002), we demonstrated that PM_10_ induced AKT phosphorylation and activation, preventing apoptosis in lung epithelial cells, with PI3K playing a critical role in the upregulation of the AKT/FoxO3a pathway under this context.

## 2. Results

### 2.1. Pre-Exposure to PM_10_ Followed by H_2_O_2_ Treatment Induced AKT and FoxO3 Phosphorylation through PI3K Activation

First, cell cultures were exposed to PM_10_ (10 µg/cm^2^), H_2_O_2_ (500 µM), or LY294002 (LY) inhibitor (50 μM), and we found that none of the concentrations tested had influence on cell viability (Table 1). The selection of the concentration used in this study was based on the dosimetric evaluation of total PM deposition in the lungs of exposed citizens from Rubidoux, California, United States. In this city, the concentration of PM is 79 µg/cm^2^ over a 24 h period. Using a dosimetry approach considering variations in airway anatomy, nasal breathing, and deposition at bifurcation points, the above-mentioned PM concentration of exposed humans reconciles with in vitro models in a PM concentration ranging from 0.2 to 20 µg/cm^2^ [21].

**Figure 1:**
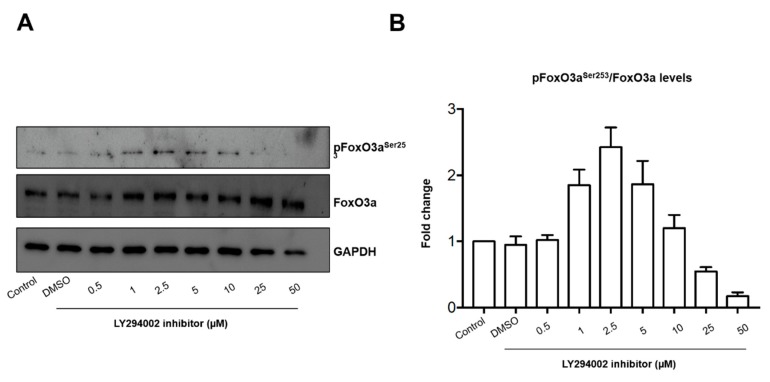
Dose-response curve of the LY294002 inhibitor over FoxO3a. The cells were incubated to a concentration of LY294002 inhibitor (0.0, 0.5, 1, 2, 5, 10, 25, 50 µM) for 24 h. Cells were cultured for 24 h in F12K medium supplemented with 10% of fetal bovine serum (FBS); Control: cells unexposed; DMSO: cells exposed only to DMSO (vehicle for LY294002). (**A**) Representative image of western blot of pFoxO3^Ser253^, FoxO3, and glyceraldehyde 3-phosphate dehydrogenase (GAPDH) as housekeeping control and (**B**) data from a duplicated assay.

**Figure 2:**
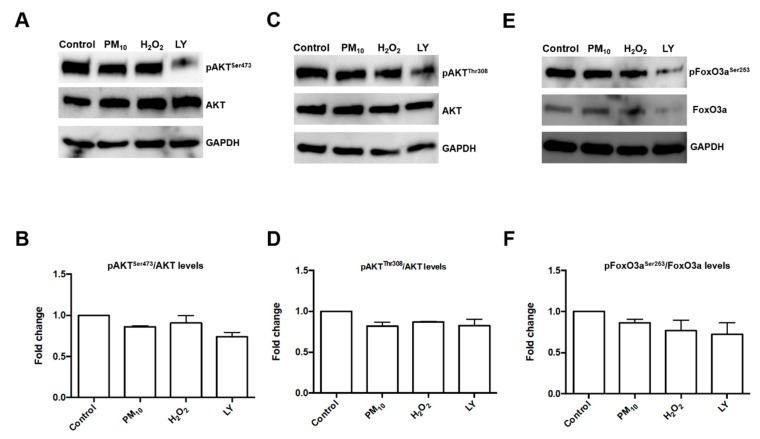
Levels of pAKT^Ser473^, pAKT^Thr308^, and pFoxO3a^Ser253^ proteins. Representative blot of (**A**) levels of pAKT^Ser473^ assessed; (**B**) densitometry of pAKT^Thr308^ protein levels using ImageJ software; (**C**) assessed pAKT^Th308^ levels; (**D**) densitometry of pAKT^Ser473^ protein levels using ImageJ software; (**E**) assessed pFoxO3a^Ser253^ levels; and (**F**) densitometry of pFoxO3a^Ser253^ protein levels using ImageJ software. Lung epithelial cells were exposed to PM_10_ (10 µg/cm2) for 24 h, treated with H_2_O_2_ (500 µM) for 24 h, and treated with LY294002 inhibitor (LY) (50 μM) for 1 h before the H_2_O_2_ (500 µM) treatment. Glyceraldehyde 3-phosphate dehydrogenase (GAPDH) protein was used as loading control for western blot. The image is representative of three independent experiments, and values are the mean ± SD of three independent experiments.

**Figure 3:**
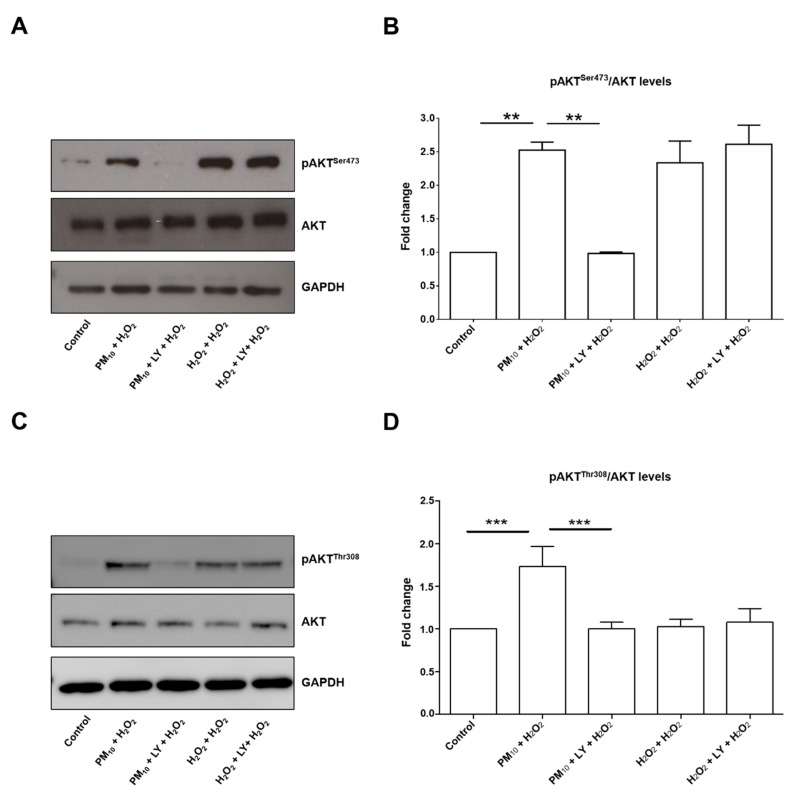
Representative blot of (**A**) pAKT^Ser473^ and total AKT, as well as (**C**) pAKT^Thr308^ and total AKT assessed by western blot and densitometry of (**B**) pAKT^Ser473^ and (**D**) pAKT^Thr308^ of levels using ImageJ software. Lung epithelial cells were pre-exposed to PM_10_ (10 µg/cm^2^) for 24 h, and then cells were treated with H_2_O_2_ (500 mM) for 24 h. In lane 3 and 6 of the blot (panel A) are cells treated with LY294002 inhibitor (50 μM) for 1 h before the H_2_O_2_ (500 µM) treatment. Glyceraldehyde 3-phosphate dehydrogenase (GAPDH) protein was used as loading control for western blot. pAKT^Ser473^ ** *p* < 0.001 versus control; ** *p* < 0.001 versus PM_10_ + H_2_O_2_ versus PM_10_ + LY + H_2_O_2_. pAKT^Thr308^ *** *p* < 0.0001 versus control; *** *p* < 0.0001 versus PM_10_ + H_2_O_2_ versus PM_10_ + LY + H_2_O_2_. The image is representative of three independent experiments, and values are the mean ± SD of three independent experiments.

The concentration of 500 µM H_2_O_2_ mimics an oxidant stimuli unable to induce cytotoxicity in lung epithelial cells [5]. The concentration of LY294002 inhibitor tested here has been used successfully for PI3K pathway inhibition in the same cell line [22]. In addition, we performed a dose-response curve for pFoxO3a^Ser253^ inhibition (Figure 1). The concentration in which we observed the inhibitory effect of LY294002 in pFoxO3a^Ser253^ was 50 µM. Our design was based in the hypothesis that a healthy population might have undetectable alterations in the respiratory tract, but a second oxidant exposure could induce damage that is undetectable after PM_10_ exposure. The second oxidant exposure could be related to infections or allergies, leading to a higher number of alterations. Specifically, we focused on a particular pathway that might partially explain the epidemiological evidence that points out the link between PM_10_ exposure and lung cancer development [2,3,23]. We have seen that PM_10_ exposure induces several alterations, including double-stranded DNA breaks, without affecting cell viability, which has raised the concern of apoptosis evasion. Since alterations in the PI3K/AKT/FoxO3a pathway have been described as cell survival mechanisms in several types of cancer, we evaluated the phosphorylation state of pAKT^Ser473^ and pAKT^Thr308^ after PM_10_ exposure and no changes were detected (Figure 2). However, a 2.5-fold and 1.8-fold increase in the pAKT^Ser473^ and pAKT^Thr308^ state, respectively, were detected in cell cultures pre-exposed to PM_10_ followed by H_2_O_2_ treatment, and this increase was completely prevented by the incubation of LY294002 inhibitor in both pAKT^Ser473^ and pAKT^Thr308^, which highlights that PI3K inhibition plays a role in the pAKT^Ser473^ state mediated by PM_10_ exposure (Figure 3). The 48 h incubation with H_2_O_2_ (500 µM) and the 48 h LY294002 inhibitor treatment in cells exposed to H_2_O_2_ (500 µM) led to similar changes in pAKT^Ser473^ (2.33-fold and 2.61-fold; Figure 3A,B). Apparently, a 48 h treatment with H_2_O_2_ (500 µM) causes a similar pAKT^Ser473^ increase, nevertheless, the LY294002 inhibitor was unable to prevent this effect, suggesting that an oxidant stimulus such as H_2_O_2_ induced and increased pAKT^Ser473^ but by an independent PI3K signaling. However, the 48 h incubation with H_2_O_2_ (500 µM) and the 48 h LY294002 inhibitor treatment in cells exposed to H_2_O_2_ (500 µM) showed no changes in the levels of pAKT^Thr308^ (Figure 3C,D).

**Figure 4:**
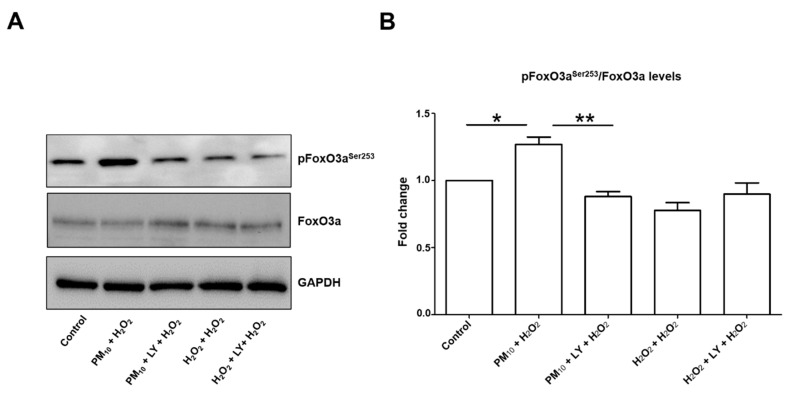
Representative blot of (**A**) pFoxO3a^Ser253^ and total FoxO3a and (**B**) densitometry of levels using ImageJ software. Lung epithelial cells were pre-exposed to PM_10_ (10 µg/cm^2^) for 24 h, and then cells were treated with H_2_O_2_ (500 mM) for 24 h. In lane 3 and 6 of the blot (panel A) are cells treated with LY294002 inhibitor (50 μM) for 1 h before the H_2_O_2_ (500 µM) treatment. Glyceraldehyde 3-phosphate dehydrogenase (GAPDH) protein was used as loading control for western blot. * *p* < 0.01 versus control; ** *p* < 0.001 versus PM_10_ + H_2_O_2_. The image is representative of three independent experiments, and values are the mean ± SD of three independent experiments.

Then, levels of FoxO3a^S253^ were assessed and found a 1.2-fold increase in cell cultures exposed to PM_10_ plus H_2_O_2_. Moreover, this increase was prevented by inhibition of PI3K using the LY294002 inhibitor (Figure 4A,B). By contrast, none of the other treatments had this increase, suggesting that PM_10_ exposure is responsible for the increase in FoxO3a^Ser253^ rate.

### 2.2. Pre-Exposure to PM_10_ Decreased Catalase and p27^kip1^ Protein through PI3K Activation

**Figure 5:**
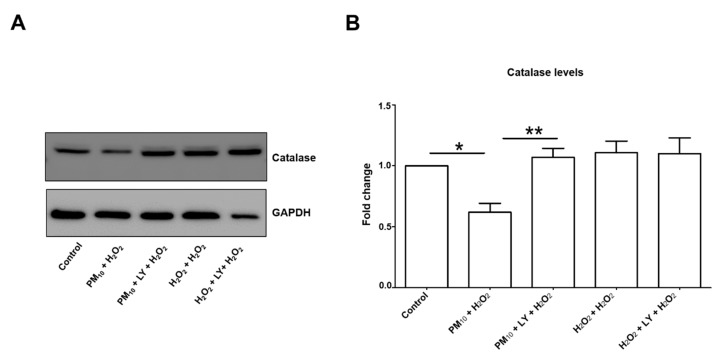
Representative blot of (**A**) assessed catalase levels and (**B**) densitometry using ImageJ software. Lung epithelial cells were pre-exposed to PM_10_ (10 µg/cm^2^) for 24 h, and then cells were treated with H_2_O_2_ (500 µM) for 24 h. In lane 3 and 6 of the blot (panel (**A**)) are cells treated with LY294002 inhibitor (LY) (50 μM) for 1 h before the H_2_O_2_ (500 µM) treatment. Glyceraldehyde 3-phosphate dehydrogenase (GAPDH) protein was used as loading control for western blot. * *p* < 0.001 versus control; ** *p* < 0.01 versus PM_10_ + H_2_O_2_. The image is representative of three independent experiments, and values are the mean ± SD of three independent experiments.

**Figure 6:**
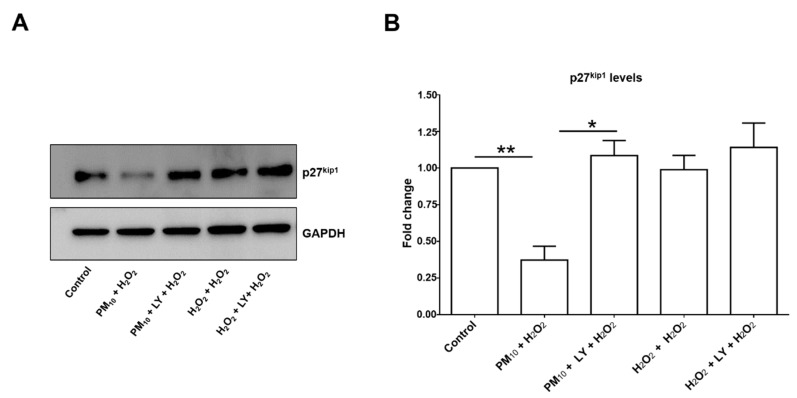
Representative blot of (**A**) assessed p27^kip1^ levels and (**B**) densitometry using ImageJ software. Lung epithelial cells were pre-exposed to PM_10_ (10 µg/cm^2^) for 24 h, and then cells were treated with H_2_O_2_ (500 µM) for 24 h. In lane 3 and 6 of the blot (panel **A**) are cells treated with LY294002 inhibitor (50 μM) for 1 h before the H_2_O_2_ (500 µM) treatment. Glyceraldehyde 3-phosphate dehydrogenase (GAPDH) protein was used as loading control for western blot. ** *p* < 0.001 versus control; * *p* < 0.001 versus PM_10_ + H_2_O_2_. The image is representative of three independent experiments, and values are the mean ± SD of three independent experiments.

Catalase and p27^kip1^ protein levels are modulated by AKT/FoxO3a (Figure 5 and Figure 6), and we found a 38.1% and 62.7% downregulation in both protein levels, respectively, in cell cultures exposed to PM_10_ followed by H_2_O_2_ (Figure 5B and Figure 6B). In both cases, PI3K inhibition completely prevented the decrease of catalase and p27^kip1^ levels, while it was unaffected by 48 h H_2_O_2_ treatment or H_2_O_2_ and LY294002 treatments (Figure 5B and Figure 6B). Interestingly, the downregulation was higher for p27^kip1^ than for catalase, which might imply that the PI3K/AKT/FoxO3a pathway has an important role in p27^kip1^ expression, while for catalase other control expression mechanisms are involved. Indeed, the number of activators and repressors reported to be involved in catalase expression has been increasing and includes SP1, NF-Y, XBP1, NRF-2, and C/EBP-β, and PPARγ and MAPK signaling, respectively, among others (Revised by Glorieux et al., 2015) [24].

### 2.3. Inhibition of Apoptosis via PI3K/AKT/FoxO3a by Pre-Exposure to PM_10_ Followed by H_2_O_2_ Treatment

**Figure 7:**
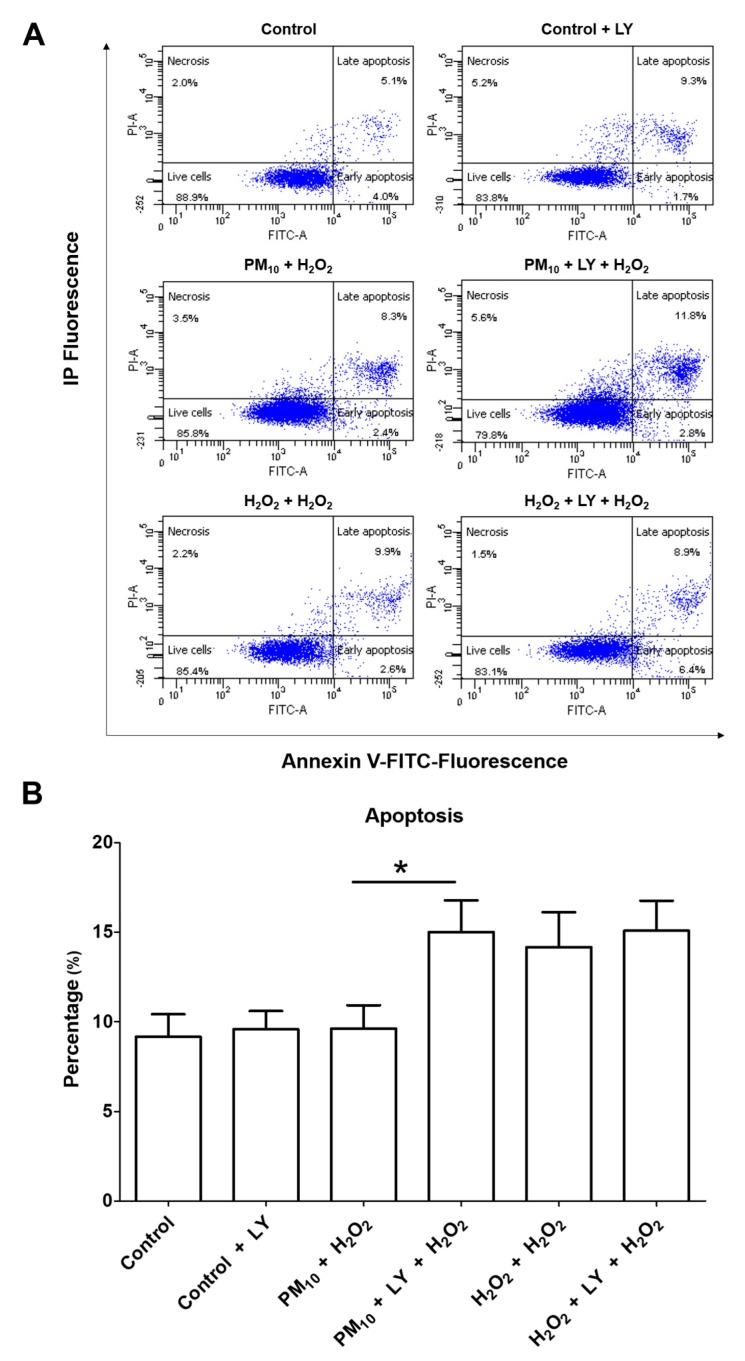
Representative plots (**A**) and graphic (**B**) of apoptosis evaluation of cells pre-exposed to PM_10_ (10 µg/cm^2^) for 24 h and then treated with H_2_O_2_ (500 mM) for 24 h. LY294002 inhibitor (LY) (50 μM) was incubated for 1 h prior to H_2_O_2_ (500 mM) treatment. (**B**) Bar graph showing the average percentage of apoptotic cells pre-exposed to PM_10_ (10 µg/cm^2^) for 24 h and then treated with H_2_O_2_ (500 mM) for 24 h. LY294002 inhibitor (50 μM; indicated in bar 2, 4, and 6) was incubated for 1 h prior to H_2_O_2_ (500 µM) treatment (lane 3 and 6). Treatments with LY294002 were incubated with LY294002 inhibitor (50 μM) 1 h before the H_2_O_2_ (500 µM) treatment. Cells were stained with Annexin V dye and propidium iodide and analyzed by a flow cytometer. * *p* < 0.01 versus PM_10_ + H_2_O_2_. The image is representative of three independent experiments, and values are the mean ± SD of three independent experiments.

Cell cultures pre-exposed to PM_10_ were treated with H_2_O_2_, and this combination had no influence on apoptosis. However, the LY294002 inhibitor revealed that these treatments had a 55.98% increase in apoptosis compared to cells exposed to PM_10_ plus H_2_O_2_ (Figure 7). Cell cultures exposed to H_2_O_2_ for 48 h had 41.8% increased apoptosis, while H_2_O_2_ for 48 h plus LY294002 inhibitor had 50.8% increased apoptosis (Figure 7), and importantly, we found that none of the concentrations tested (PM_10_ (10 µg/cm^2^), H_2_O_2_ (500 µM), or LY294002 (LY) inhibitor (50 μM)) had influence on cell viability (Table 1).

This result reveals that PM_10_ exposure activates the PI3K/AKT/FoxO3a pathway, which prevents cell death after the second oxidant challenge. The outcome of forced apoptosis evasion induced by PM_10_ + H_2_O_2_ exposure might lead to replication of lung epithelial cells with unrepaired DNA. Unfortunately, the apoptosis resistance plays a central role in carcinogenesis, tumor development and progression, and chemotherapy resistance. In addition, replication of these cells could be a key point in which newly divided cells acquire a cancer-like phenotype [25].

## 3. Discussion

In this study, we demonstrated that PM_10_ exposure had no influence on the PI3K/AKT/FoxO3a pathway in lung epithelial A549 cells. However, cell cultures previously exposed to PM_10_ were challenged to H_2_O_2_, which induced an upregulation in AKT (pAKT^Ser473^ and pAKT^Thr308^)/pFoxO3a^Ser253^. On one hand, H_2_O_2_ would mimic a prooxidant environment that can be reached in the respiratory tract by bacterial or viral infections [26,27], and on the other hand, the deregulation of AKT/FoxO3a signaling has a central role during cellular disturbances found in diseases such as leukemia [28], diabetic kidney disease [29], and breast cancer [30], among others.

Specifically, to investigate whether PM_10_ exposure could activate the above-mentioned pathway, we decided to use 2-(4-morpholinyl)-8-phenyl-4H-1-benzopyran-4-one, which is commercially known as LY294002. This compound inhibits purified PI3K without inhibition of other kinases or enzymes requiring ATP [31]. This compound is an analog of quercetin, used in the past as an unspecific kinase inhibitor; however, quercetin acts as an inhibitor at IC50 = 3.8 µM while LY294002 inhibitor displays an IC50 = 1.8 µM. Based on this information, LY294002 has been widely used as a PI3K inhibitor in in vivo [32] and in vitro models, including cortical neurons [31] and lung epithelial A549 cells [22]. LY294002, a reversible inhibitor of PI3K, and wortmannin, an irreversible inhibitor of PI3K, are the most described and used PI3K inhibitors [31,33,34]. Although both are considered as specific and invaluable tools to study the PI3K pathway, the inhibitory profile of LY294002 is broader than wortmannin. LY294002 inhibit mTOR (mammalian target of rapamycin), DNA-PK (DNA-dependent protein kinase), as well as other kinases, such as CK2 (casein kinase 2) and Pim-1 [35,36,37]. Also, LY294002 activates AKT and accumulate phospho-AKT at the intracellular membrane, a condition that is abolished by treatment with wortmannin [38]. Thus, the effects in apoptosis evasion by the combination of PM_10_ and H_2_O_2_ can be prevented by the non-selective inhibitor of PI3K selected for our study, displaying an effect associated to cell survival in cancer cells.

To our knowledge, we demonstrate for the first time that PM_10_ exposure followed by an oxidant stimulus with H_2_O_2_ upregulates the pAKT^Ser473^/pAKT^Thr308^/pFoxO3a^Ser253^, with a reduction in apoptosis mediated by PI3K in lung epithelial cells, which could mimic air pollutants exposure followed by oxidant endogenous stimulus seen, for instance, during bacterial infections. Nevertheless, a 65% downregulation of FoxO3a protein levels in the entire lung tissue from mice exposed to PM_2.5_ (17.7 μg/m^3^) inhalation over 12 weeks has been previously reported [39]. However, the rate of phosphorylation was undetermined, and the quantification performed showed the global FoxO3a levels, whereas our study reveals the response of a specific cell lineage. In addition, FoxO3a^Ser253^ upregulation was only found after H_2_O_2_ was used as a second oxidant stimulus, which suggests that PM_10_ predisposes to deregulation in AKT/FoxO3a signaling that could evade apoptosis.

We also found that AKT (pAKT^Ser473^ and pAKT^Thr308^)/pFoxO3a^Ser253^ activation was associated with a decrease in catalase protein, leading to a reduction in the antioxidant defense since this enzyme is responsible for H_2_O_2_ detoxification [40]. Thus, cells with downregulated catalase might accumulate higher amounts of H_2_O_2_ that would lead to a highly oxidant environment. In addition, H_2_O_2_ is the precursor of hydroxyl radical, a well-known species with strong DNA affinity that has no enzymatic defense for detoxification. Indeed, we have already demonstrated that PM_10_ exposure induced a decrease in the antioxidant enzymatic activity of catalase, superoxide dismutase, glutathione reductase, and glutathione S-transferase without affecting cell death, and this effect was not reverted by hydroxyl radical chelation [5], which highlights that PM_10_ induces irreversible enzymatic activity damage in lung epithelial cells.

The decrease of p27^kip1^ levels in this study suggests a deregulation in the progression from the G_1_ to the S phase of the cell cycle that can be associated with the inhibition of apoptosis mediated by the AKT (pAKT^Ser473^ and pAKT^Thr308^)/pFoxO3a^Ser253^ pathway activated by PM_10_ exposure in combination with H_2_O_2_ in lung epithelial cells. However, what is concerning is the strong evidence of decrease or loss of p27^kip1^ in several tumors, including breast, colon, and prostate adenocarcinomas [20]. In addition, Liu and colleagues recently suggested that some cell-cycle regulators exert important independent-cycle functions, and p27^kip1^ is listed in their work, emphasizing that this protein has a role in postmitotic neurons and immune T cells [41]. Therefore, deregulation of p27^kip1^ levels could have an impact on pulmonary T cells [42] and immune cells from bloodstream [43,44] and brain [45]. Perhaps exposure solely to PM_10_ is not enough to induce deregulation of some proteins such as p27^kip1^, but a second oxidant challenge could trigger tissue dysfunction. Besides infections as a possible second oxidant challenge, H_2_O_2_ is detected in exhaled breath of patients with non-small cell lung cancer but also in healthy cigarette smokers [46], which highlights the possibility that PM_10_ exposure can really be followed by a H_2_O_2_ oxidant environment with unknown consequences.

Regarding the PM_10_ exposure model, findings of this study represent the effect of the complete particle (PM_10_) with all the organic and non-organic components, and the contribution of each component cannot be dissected. However, the link between lung cancer and PM_10_ exposure is attributed to the inhalation of the entire particle plus smaller fractions. Importantly, because PM_10_ is derived from anthropogenic activity and each city has non-identical PM_10_ chemical compositions, a different apoptotic evasion footprint would be expected according to the city where the PM_10_ is located. In this case, PM_10_ collected from Mexico City is derived from traffic emissions from 5,000,000 vehicles, volatile organic compounds derived from thinners, degreasers, cleaners, lubricants, and liquid fuels used in shops like drycleaners, and liquefied petroleum gas leaks in houses, while metals come from the industrial sector located on the north side of Mexico City. Unfortunately, wind transports other contaminants from adjacent cities to Mexico City’s atmosphere.

Based on the IARC Scientific Publication No. 161 published in 2013, a complete PM_10_ particle is spatially and temporally heterogeneous, which means that there is geographical variability of composition and concentration among the PM analyzed from Europe, Africa, and the United States. However, epidemiological studies clearly show an association between PM exposure and the risk of cancer, while the experimental research provides a basis for the plausibility of a risk of cancer regardless of the city in which the PM was collected. Beyond the chemical heterogeneity, air pollution’s classification as a group 1 carcinogen is attributed to the fact that some carcinogenic or highly toxic compounds derived from fuel combustion are common, regardless of the combustion source. For instance, some countries can have higher fuel combustion for industrial purposes, whereas others might have combustion for heating, cooking, and heavy automobile traffic, but these sources might generate formaldehyde, acrolein, benzene, toluene, 1,3-butadiene, benzo[a]pyrene, iron, and sulfates, among others.

We recognize that the findings of this study were identified in A549 cells, which are already transformed cells. Nevertheless, if this cell line was able to display such important alterations, perhaps normal lung epithelial cells might have higher susceptibility to stress induced by PM_10_ exposure followed by oxidant stimulus. Indeed, even if the results can only be restricted to lung epithelial cells, we cannot discard the notion that a similar response could be found in bronchial cells, which are also targeted by PM_10_ exposure.

Finally, we cannot dismiss that other apoptosis-evasion mechanisms could be involved and were not explored in this study. For instance, the mTOR pathway, mitochondrial disturbances, and immune cell inactivation could act together at the tissue level to explain why PM_10_ exposure is classified as carcinogenic to humans. In addition, early PM_10_ exposure time points must be investigated because PI3K activation is an immediate cellular response. As an example, viral infection activates pAKT^Ser473^ after 15 min with a sustained increase until 4 h [47]. Besides, it is probable that the unseen decrease of pAKT^Ser473^ levels could be related to negative regulatory feedbacks after PI3K inhibition with LY294002. For example, it has been reported that insulin feedback induced by PI3K inhibitors could reactivate the PI3K-mTOR signaling in tumors [48]. Further studies considering a time course evaluation of pAKT^Ser473^ under the context of pre-exposure of PM_10_ and treatment with H_2_O_2_ need to be performed in order to elucidate if treatment with LY294002 is capable of reduce AKT phosphorylation.

## 4. Materials and Methods

### 4.1. PM_10_ Sampling

PM_10_ was collected in a residential-urban area in Mexico City. A large volume particle collector was used with 1.13 m^3^/min flow (GMW model 1200 VFCHVPM10 Sierra Andersen, Smyrna, GA, USA) and equipped with cellulose filters (Sartorius AG, Goettingen, Germany). After collection, the nitrocellulose filters were kept in a desiccator at 4 °C in the dark. The particles were recovered from the membranes and stored in a vial of glass, sterile and free of endotoxins. The PM_10_ was kept at 4 °C in a desiccator and in the dark until used [49].

### 4.2. Lung Epithelial A549 Cell Culture

The lung epithelial A549 cell line derived from a human lung adenocarcinoma used in this work was obtained from the American Type Culture Collection (CCL-185; ATCC, Manassas, Virginia, United States). Cell cultures were grown with F12 Kaighn’s culture medium (21127-022; Gibco, Grand Island, New York, United States) supplemented with 10% fetal bovine serum (FBS;16000-044; Gibco, Carlsbad, California, United States) at 37 °C and in a 5% CO_2_ atmosphere. The cells were growing to 70% confluence in early passages.

### 4.3. PM_10_ Exposure, H_2_O_2_ Treatment, and LY294002 Inhibitor in Cell Cultures

The experiments were carried out in 6-well plates; 125,000 cells were seeded and after 24 h, cell cultures were exposed to the different treatments. For PM_10_ exposure, 95 µg of PM_10_ (solid particles) were weighed in a sterilized and free-endotoxin vial. Then, 200 µL of F12 Kaighn’s medium supplemented with 10% FBS were added to the vial of particles and gently mixed by pipetting for 10 s. Next, this suspension (200 µL containing the PM_10_ particles) was added to a 6-well plate with seeded cells, which had 1800 µL of F12 Kaighn’s medium supplemented with 10% FBS in each well. The area of each well from the 6-well plate is 9.5 cm^2^; therefore, the final PM_10_ concentration is 10 µg/cm^2^. Cells were exposed to PM_10_ for 24 h with this procedure when PM_10_ treatment is indicated in this study. For combination with H_2_O_2_, the culture medium was removed, and cells were washed with PBS and treated with 500 µM H_2_O_2_ (TA-125-HP; Thermo Fisher, Fremont, California, United States) for 24 h. In addition, we used LY294002 (9901S; Cell Signaling, Danvers, Massachusetts, United States) as an inhibitor of the AKT/Fox3a pathway at 50 μM in DMSO before the H_2_O_2_ treatment. The LY294002 concentration was determined through a dose-response curve over inhibition of pFoxO3a^Ser253^. Briefly, cells A549 were cultured in F12 Kaighn’s medium supplemented with 10% of FBS. After 24 h, the cells were incubated with LY294002 inhibitor (0.0, 0.5, 1, 2, 5, 10, 25, and 50 µM) for 24 h, then cells were lysed (20 mM Tris, 150 mM NaCl, 1% NP-40) using phosphatase and protease inhibitors (78440; Thermo Fisher, Rockford, Illinois, United States). The inhibitory effect of LY294002 over pFoxO3a^Ser253^ was evaluated through western blot of pFoxO3^Ser253^, FoxO3a, and glyceraldehyde 3-phosphate dehydrogenase (GAPDH) as housekeeping control (two independent experiments). Control group was supplemented with DMSO in order to evaluate the effect caused by treatments and avoid any bias in the experimental design. Treatments labeled as H_2_O_2_ + H_2_O_2_ were incubated for 24 h with 500 µM H_2_O_2_, then cells were extensively washed with cell culture medium without serum, and, again, cells were treated with 500 µM H_2_O_2_ for 24 h.

### 4.4. Cellular Viability

After cell cultures were exposed to the different treatments (Section 4.3), cell viability was evaluated using the trypan blue dye exclusion assay. Briefly, the cells’ exposure to different treatments were collected using 0.25% trypsin-EDTA (25200055; Thermo Fisher, Carlsbad, California, United States) and dyed with trypan blue (0.4%), and then 500 cells were counted using an inverted microscope (Leica). Cells that did not incorporate the trypan blue dye were considered viable cells. The results are presented as the percentage of cells from three independent experiments.

### 4.5. Determination of Protein Levels

After treatments, cell cultures were lysed (20 mM Tris, 150 mM NaCl, 1% NP-40) and phosphatase and protease inhibitors (78440; Thermo Fisher, Rockford, Illinois, United States) were added. Protein content was analyzed using the bicinchoninic acid reagent (SIGMA: B9643) using bovine serum albumin as standard curve. The principle is based on the reduction of copper (2+) to copper (1+) by proteins, and then copper (1+) reacts with bicinchoninic acid that absorbs light at 562 nm [50]. The protein content in lysates from cell cultures was compared with a standard solution of bovine serum albumin (23209; Thermo Fisher, Carlsbad, California, United States).

Then, 30 µg of protein was loaded in a 12% SDS-PAGE for detection of AKT, as well as phosphorylated pAKT^Ser473^ and pAKT^Thr308^ (2920S, 4060S, and 13038S, respectively; Cell Signaling, Danvers, Massachusetts, United States), the transcription factor FoxO3a (ab12162; Abcam, Cambridge, Massachusetts, United States) and phosphorylated pFoxO3a^Ser253^ (PA5-36816; Thermo Fisher, Rockford, Illinois, United States), and finally catalase and p27^kip1^ (12098S, and D69C12, respectively; Cell Signaling, Danvers, Massachusetts, United States). The proteins were transferred to a polyvinylidene fluoride (PVDF) membrane using a semidry blotting system (Trans-Blot-Turbo, transfer system; Bio-Rad, Hercules, California, United States) at 25 V for 30 min. PVDF membranes were blocked (5% low-fat milk in 0.1% Tween 20 in TBS) at room temperature for 1 h, then washed 3 times with 10 mL of 0.1% Tween 20 in TBS. Later, PVDF membranes were incubated overnight with primary antibodies in 5% low-fat milk, as follows: anti-pAKT^Ser473^ (dilution 1:2000), anti-pAKT^Thr308^ (dilution 1:2000), anti-AKT (dilution 1:2000), anti-pFoxO3^Ser253^ (dilution 1:1000), anti-FoxO3a (dilution 1:1000), anti-catalase (dilution 1:2000), anti-p27^kip1^ (dilution 1:1000), and anti-GADPH (dilution 1:1000). The membranes were incubated with mouse or rabbit secondary antibodies as appropriate, coupled to horseradish peroxidase. Finally, the membranes were revealed using a chemiluminescent reagent (WBKLS0100; Millipore, Hertfordshire, Watford, United Kingdom) and visualized using a UVP luminescence reader. Protein levels were evaluated using densitometry through Image J software (rsb.info.nih.gov/ij) [51].

### 4.6. Apoptosis Measurement

The apoptosis was determined by staining cells with Annexin V-fluorescein isothiocyanate (Ex/Em at 495 nm/529 nm) [52]. A549 cells (8 × 10^5^/well) were seeded in plates of 6-well culture slides with F12K medium supplemented with 10% FBS for 24 h. After the cells were treated as described in Section 4.3, they were washed with PBS and centrifuged at 200 *g* for 5 min. The pellet obtained was resuspended in 100 µL of Annexin V solution (Annexin V FLUOS dying kit, 1858777; Roche Diagnostics GmbH, Mannheim, Germany) and incubated 15 min at 15 °C. FACSDiva software v.1.1 was used for data acquisition and analysis. The early and late apoptosis were considered and were used as control undyed cells and cells dyed with Annexin-V and propidium iodide.

### 4.7. Statistical Analysis

The results are presented as the means ± standard deviations of three independent experiments. The statistical analysis of variance was applied with the multiple comparisons test with the Prism Program version 5 (GraphPad Software). A value of *p* < 0.05 was considered statistically significant.

## 5. Conclusions

In this study, we demonstrated that lung epithelial A549 cells pre-exposed to PM_10_ had higher susceptibility to developing an upregulation of the AKT/FoxO3a pathway mediated by PI3K activity that leads to apoptosis evasion when cells are challenged to a second oxidant stimulus, such as H_2_O_2_.

## Figures and Tables

**Table 1:** Cell viability of lung epithelial A549 cells exposed to outdoor particulate matter with aerodynamic size ≤ 10 µm (PM_10_) and H_2_O_2_.

Control	LY294002	PM_10_+H_2_O_2_	PM_10_+LY+H_2_O_2_	H_2_O_2_+H_2_O_2_	H_2_O_2_+LY+H_2_O_2_
100	95.23 (SD ± 15.2)	108.8 (SD ± 7.7)	103.7 (SD ± 3.2)	83.3 (SD ± 3.2)	82 (SD ± 7.9)

Control: cells cultured during 24 h with free fetal bovine serum, washed and replaced with fresh cell culture medium, and incubated for a second period of 24 h. LY294002: Cells exposed to 50 µM of LY294002 inhibitor for 48 h. PM_10_ + H_2_O_2_: cells exposed to PM_10_ for 24 h and exposed 500 µM H_2_O_2_ for 24 h. PM_10_ + LY + H_2_O_2_: cells pre-exposed to 10 µg/cm^2^ PM_10_, incubated with 50 µM LY294002 (inhibitor of PI3K), and treated with 500 µM H_2_O_2_. H_2_O_2_ + H_2_O_2_: cells exposed to 500 µM H_2_O_2_ during 24 h, washed with cell culture medium and treated again with H_2_O_2_. H_2_O_2_ + LY + H_2_O_2_: cells exposed to H_2_O_2_ during 24 h and treated with LY294002 and exposed to H_2_O_2_. The results are the mean ± SD of three independent experiments.
